# Targeting a novel domain in podoplanin for inhibiting platelet-mediated tumor metastasis

**DOI:** 10.18632/oncotarget.6598

**Published:** 2015-12-14

**Authors:** Takaya Sekiguchi, Ai Takemoto, Satoshi Takagi, Kazuki Takatori, Shigeo Sato, Miho Takami, Naoya Fujita

**Affiliations:** ^1^ Division of Experimental Chemotherapy, Cancer Chemotherapy Center, Japanese Foundation for Cancer Research, Tokyo, Japan; ^2^ Department of Computational Biology and Medical Sciences, Graduate School of Frontier Sciences, The University of Tokyo, Japan

**Keywords:** podoplanin, platelets, platelet aggregation, neutralizing antibody, tumor metastasis

## Abstract

Podoplanin/Aggrus is a sialoglycoprotein expressed in various cancers. We previously identified podoplanin as a key factor in tumor-induced platelet aggregation. Podoplanin-mediated platelet aggregation enhances tumor growth and metastasis by secreting growth factors and by forming tumor emboli in the microvasculature. Thus, precise analysis of the mechanisms of podoplanin-mediated platelet aggregation is critical for developing anti-tumor therapies. Here we report the discovery of a novel platelet aggregation-inducing domain, PLAG4 (81-EDLPT-85). PLAG4 has high homology to the previously reported PLAG3 and contributes to the binding of its platelet receptor CLEC-2. Mutant analyses indicated that PLAG4 exhibits a predominant platelet-aggregating function relative to PLAG3 and that conserved Glu^81^/Asp^82^/Thr^85^ residues in PLAG4 are indispensable for CLEC-2 binding. By establishing anti-PLAG4-neutralizing monoclonal antibodies, we confirmed its role in CLEC-2 binding, platelet aggregation, and tumor emboli formation. Our results suggest the requirement of simultaneous inhibition of PLAG3/4 for complete suppression of podoplanin-mediated tumor growth and metastasis.

## INTRODUCTION

Tumor cell growth and survival are affected by a wide variety of tumor microenvironments. During hematogenous metastasis processes, circulating tumor cells rarely survive due to exclusion by the immune system. Platelets are known as the critical component that affects the survival of circulating tumor cells, leading to metastasis formation [1]. In fact, anti-platelet agents and thrombocytopenia reduce tumor metastasis in experimental models [2, 3]. Moreover, the administration of anti-coagulants has been reported to lower the mortality rate [4, 5]. The following mechanisms underlying platelet-promoting metastasis are proposed: (i) enhancement of tumor cell embolization in the microvasculature by the formation of large tumor cell-platelet aggregates, (ii) up-regulation of tumor malignancy by the release of soluble factors from activated platelets, and (iii) protection from immunological assault or blood shear stress by coating of the tumor cell surface [1]. As all pathways are triggered by tumor–platelet interaction, this interaction could be a promising target for cancer therapy.

We have previously identified a type-I transmembrane sialoglycoprotein, podoplanin, also known as Aggrus, as a platelet aggregation-inducing factor in highly metastatic tumor cells [6]. Podoplanin expression induces platelet aggregation, and experimental and spontaneous pulmonary metastasis [3, 6]. Podoplanin is frequently overexpressed in various malignant tumors such as squamous cell carcinoma, mesothelioma, glioblastoma, bladder tumors, and osteosarcoma [7–11]. Therefore, the platelet-interaction and the platelet-aggregating ability of podoplanin may be a target for suppressing metastasis in clinical situations.

Podoplanin contains three tandemly repeated EDXXVTPG motifs in the extracellular domain. Analysis of the epitope of a neutralizing anti-mouse podoplanin monoclonal antibody (mAb) 8F11 helped to identify the domains critical for exhibiting platelet-aggregating ability. Therefore, we designated the motif-containing domain (EDXXVTPG; in which “X” may be any amino acid) as the PLatelet AGgregation-stimulating (PLAG) domain (PLAG1–3) [6]. These domains are highly conserved among mammals in a triplicated manner. The PLAG1 and/or PLAG3 domain contains a predicted glycosylated Thr residue that is critical for podoplanin activity [3, 6, 12]. *O*-glycanase treatment reduces podoplanin's platelet aggregation ability [13], and studies using a series of glycosylation-deficient CHO cell mutants and genetically modified yeast suggest the requirement of sialylated *O*-glycan for podoplanin-induced platelet aggregation [14, 15]. In fact, a disialyl-core structure on Thr^52^ in PLAG3 has been detected in human podoplanin [16].

The C-type lectin-like receptor 2 (CLEC-2), originally identified as a platelet receptor for the snake venom toxin rhodocytin [17], has been identified as a podoplanin receptor [18]. Podoplanin binding to CLEC-2 transmits platelet-activation signals through Src family kinases, Syk, and phospholipase Cγ2 in platelets [17, 19]. CLEC-2-deficient platelets can still respond to platelet agonists, such as collagen and ADP, suggesting the possibility that the interfering podoplanin–CLEC-2 interaction may not affect physiological hemostasis [20]. Till date, we and another group have generated several PLAG domain-recognizing anti-human podoplanin mAbs that suppress podoplanin binding to CLEC-2, platelet aggregation, and hematogenous metastasis [21–23]. Because our generated anti-podoplanin neutralizing mAbs, P2–0 and MS-1, recognized Gly^45^ in PLAG2 and Asp^48^ and Asp^49^ in PLAG3, the perimeter structure around PLAG2 and PLAG3 could be the CLEC-2-binding site [22, 23]. A recent report on the crystal structure of human podoplanin-derived *O*-glycosylated PLAG2–PLAG3 peptide in complex with CLEC-2 extracellular domain revealed that the acidic side chains of Glu^47^, Asp^48^, and the sialic acid attached to Thr^52^ on PLAG3 were recognized by basic residues on CLEC-2 [24].

Here we report the discovery of an additional PLAG domain (81–85 aa in human podoplanin), which is highly conserved among mammals. The region includes the highly conserved EDXXT motif, which is closely related to the PLAG domain consensus sequence EDXXVTPG. Deletion of the region or a point mutation in it drastically attenuated its binding to CLEC-2 and podoplanin-induced platelet aggregation. Thus, we designated the region as the fourth PLAG domain, PLAG4. Importantly, deletions or point mutations in both PLAG3 and PLAG4 domains almost completely suppressed the CLEC-2-binding ability and platelet-aggregating activity of human podoplanin, whereas the deletion of or point mutation in either one of the two domains could not completely suppress the binding ability and platelet-aggregating ability. To further investigate the requirement of the PLAG4 domain for podoplanin's platelet-aggregating ability, we tried to establish PLAG4-recognizing mAbs. The generated PLAG4-recognizing mAbs exhibited CLEC-2-binding inhibitory activity and platelet aggregation-neutralizing ability. Moreover, the injection of the mAbs suppressed podoplanin-mediated hematogenous metastasis *in vivo*. These results suggest that the newly identified PLAG4 domain plays a critical role in the binding to CLEC-2, hence being a promising target motif for suppressing podoplanin-expressing tumor cell growth and metastasis.

## RESULTS

### Identification of a novel CLEC-2-binding domain on human podoplanin

**Figure 1:**
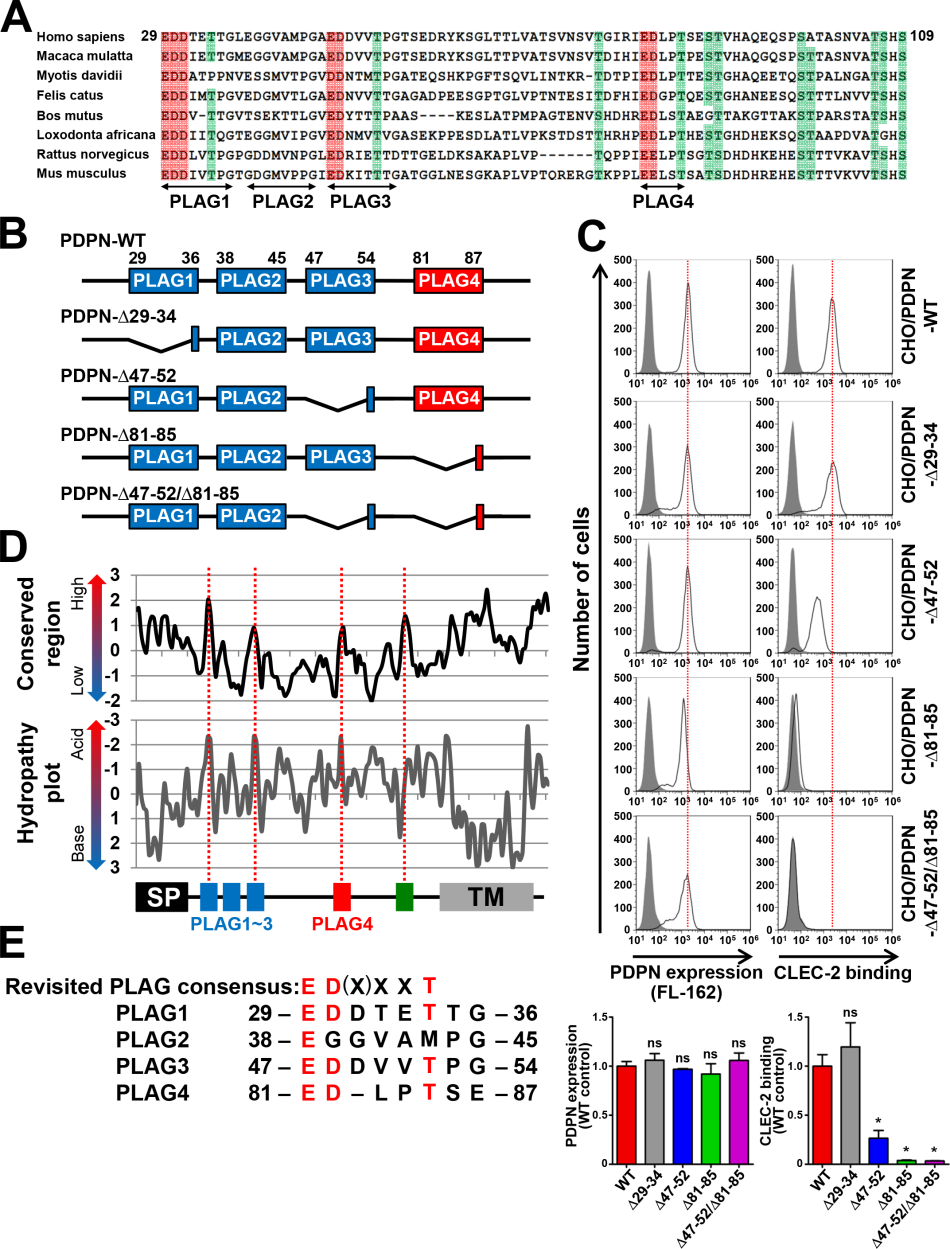
Identification of a new CLEC-2-binding domain, PLAG4, highly conserved in mammals (**A**) Eight mammalian podoplanin protein sequences were aligned. Half-tone meshing area indicates over 80% conserved residues (Red: Asp or Glu, Green: Thr or Ser). The gaps against the Homo sapiens podoplanin found in multi-aligned sequences were deleted to show the alignment concisely. The deleted gaps in each sequence of species are followed. Macaca mulatta (XP_001106933.2), not deleted; Myotis davidii (XP_006766770.1), ΔA129; Bos mutus (XP_005889851.1), ΔP100-P112; Felis catus (XP_006934362.1), ΔT63; Loxodonta africana (XP_010591406.1), ΔT63 and ΔH92; Rattus norvegicus (NP_062231.1), ΔT63; and Mus musculus (NP_034459.2), ΔT63. (**B**) Schematic representation of human PLAG domain-deleted mutants used in this study. (**C**) CHO cells that had been stably transfected with PDPN-WT or PLAG domain-deleted PDPN mutants were treated with control rabbit IgG (closed areas) or anti-PDPN pAb (FL162; open areas) for measuring PDPN expression levels (upper left panels), or with PBS (closed areas) or CLEC-2-(His)_10_ (open areas) for estimating CLEC-2-binding ability (upper right panels). After washing, cells were incubated with Alexa Flour 488-conjugated second antibody. The flow cytometry data (upper) and their quantitative graphs (lower) are shown. Each value in the lower graphs indicates mean ± SDs (*N* = 3) of the peak values normalized by that of PDPN-WT/CHO. *P < 0.05 using Mann–Whitney *U* test. ns, not significant. (**D**) Sliding-window analysis and hydropathy analysis were performed using data from 42 mammalian species (window size equals three or two amino acids, respectively). Four highly conserved regions within the extracellular domains are indicated by red dotted lines. (**E**) Human PLAG1–4 domains were aligned with the revised PLAG consensus sequence ED(X)XXT.

We previously identified that podoplanin contains three tandemly repeated PLAG domains (PLAG1–3, Figure 1A) that are critical to its platelet aggregation-inducing ability [6]. In addition, glycosylations on podoplanin are reported to be essential for its platelet aggregation-inducing ability [14, 15]. To evaluate their contributions to CLEC-2 binding, we estimated the recombinant CLEC-2 binding to CHO cells that had been transfected with a PLAG1- or PLAG3-deleted *podoplanin* cDNA-containing plasmid. We generated a PLAG1-deletion mutant by deleting the 29–34 aa-coding region and a PLAG3-deletion mutant by deleting the 47–52 aa-coding region (Figure 1B). We confirmed that the expression level of wild type (WT) or deleted podoplanin was almost the same among the transfectants (Figure 1C, left panels). Surprisingly, the Δ29–34/PLAG1 deletion did not affect the binding of podoplanin to CLEC-2 (Figure 1C, right panels). Interestingly, the deletion of Δ47–52/PLAG3 could not abrogate podoplanin binding to CLEC-2 but only showed a partial reduction of its binding capability (Figure 1C, right panels). These results suggest that other regions in podoplanin may be associated with the binding to CLEC-2. We therefore analyzed the highly conserved regions of mammalian podoplanin amino acid sequences (Figure 1D). Sequences of 42 mammalian species retrieved from the NCBI Reference Sequence Database were selected (Supplementary Figure S1), and data were analyzed using sliding-window analysis and hydropathy plots (Figure 1D). Apart from the N-terminal signal peptide, we found four highly conserved regions within the extracellular domain (red dotted lines in Figure 1D). Three out of four regions contained highly negative-charged motifs, and the forth conserved region did not (hydropathy plots in Figure 1D). We studied them in detail and found that the three acidic regions were composed of two negatively charged amino acids followed by a Thr residue (Figure 1E) and that the forth region contained a completely different conserved sequence TSHS (106–109 aa). Consequently, the first region was identified as the PLAG1 domain, the second region was located in the PLAG3 domain, and the third region was located in the middle region (81–85 aa). Because no analysis of the third region had been carried out thus far, we further analyzed its role in CLEC-2 binding and platelet aggregation. We established CHO cells that had been transfected with Δ81–85-podoplanin and examined its ability to bind to CLEC-2 (Figure 1B and 1C). Surprisingly, the deletion of 81–85 aa attenuated the CLEC-2-binding ability more than the Δ47–52/PLAG3 deletion, and the double deletion of Δ47–52/PLAG3 and Δ81–85 almost completely suppressed the binding capability. Deletion of 81–85 aa residues did not affect the membrane localization or expression level (Figure 1C). Thus, we speculated that this locus was associated with CLEC-2 binding, similarly to our previously reported PLAG domain. We therefore designated the region as the “PLAG4” domain (Figure 1E).

### Critical roles of PLAG4 domain in CLEC-2 binding and platelet aggregation

**Figure 2:**
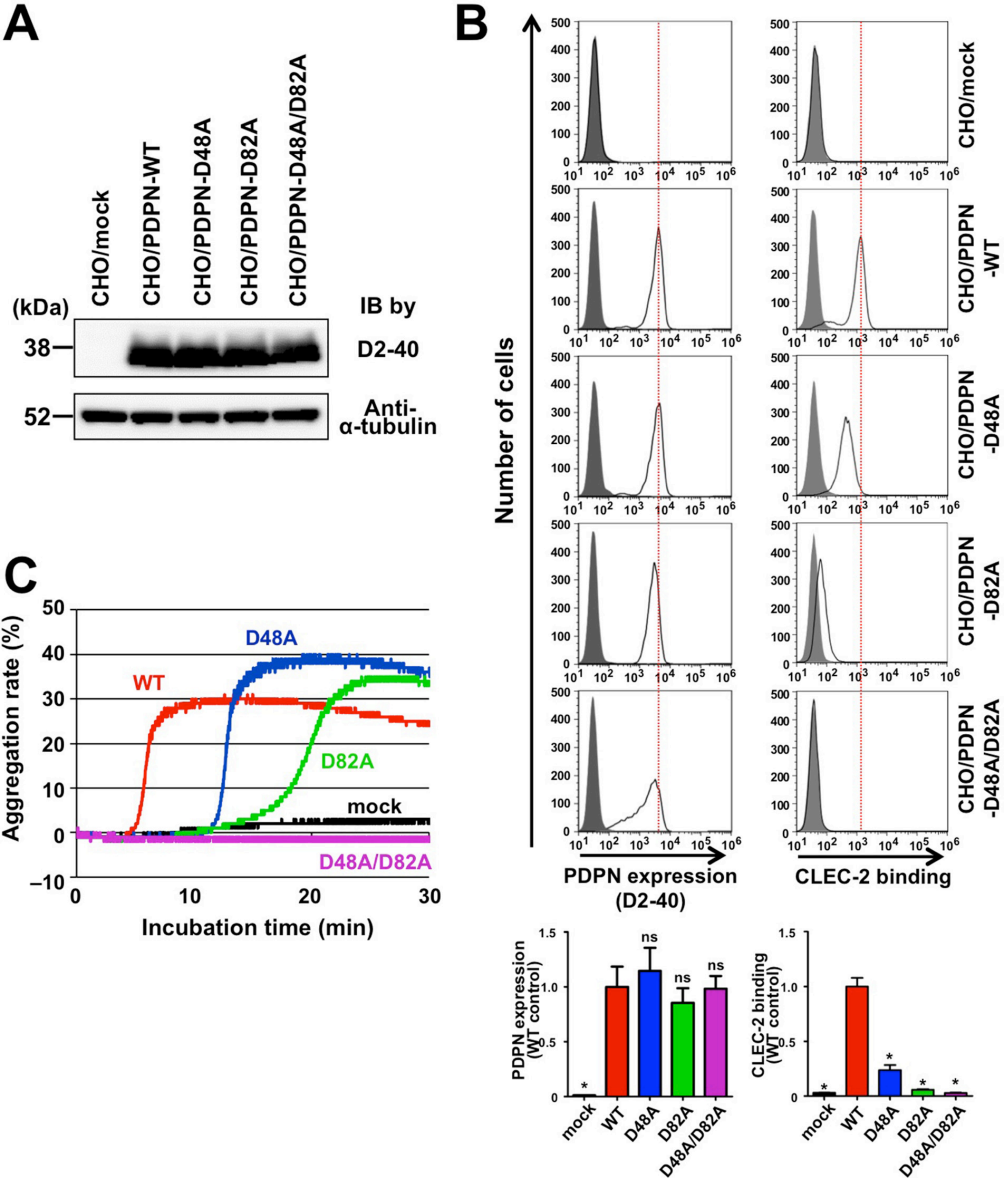
Involvement of PLAG4 domain in CLEC-2 binding and platelet aggregation (**A**) CHO cells that had been stably transfected with empty vector (mock), WT-PDPN, or PLAG3/4 point mutants were lysed and immunoblotted with antibodies to podoplanin (D2–40) or α-tubulin. (**B**) CHO cells that had been stably transfected with empty vector (mock), WT-PDPN, or PLAG3/4 point mutants were treated with control mouse IgG (closed areas) or anti-PDPN mAb (D2–40; open areas) for checking PDPN expression level (upper left panels), or with PBS (closed areas) or CLEC-2-(His)_10_ (open areas) for estimating CLEC-2-binding ability (upper right panels). After washing, cells were incubated with Alexa Flour 488-conjugated second antibody. The flow cytometry data (upper) and their quantitative graphs (lower) are shown. Each value in the lower graphs indicates mean ± SDs (*N* = 3) of the peak value normalized by that of PDPN-WT/CHO. *P < 0.05 using Mann–Whitney *U* test. ns, not significant. (**C**) CHO cells that had been stably transfected with empty vector (mock), WT-PDPN (WT), or PLAG3/4 point mutants (D48A, D82A, and D48A/D82A) were incubated with mouse PRP. The aggregation rate was estimated using an aggregometer.

To exclude the possibility that the deletion of domains affected 3D conformation, potentially leading to changes in the CLEC-2 interaction surface, we tried to generate human podoplanin point mutants exhibiting low affinity to CLEC-2. Our previously established neutralizing anti-human podoplanin mAbs, P2–0 and MS-1, interfere with the binding of human podoplanin to CLEC-2, recognizing the perimeter structure around Gly^45^, Asp^48^, and Asp^49^ residues over PLAG2 and PLAG3 domains in human podoplanin [22, 23] (Supplementary Figure S2A). Because the Asp^48^ residue is an amino acid that is critical for the recognition by anti-PLAG3 mAbs (Supplementary Figure S2A) and its acidic side chain is suggested for the binding to CLEC-2 [24], we expected that the highly conserved Asp^48^ residue in the PLAG3 domain would play an important role in the interaction with CLEC-2. In fact, the substitution of Asp^48^ residue in PLAG3 to Ala (D48A) reduced the CLEC-2 binding, and compared with the D48A, the substitution of the Asp^82^ residue in PLAG4 to Ala (D82A) partially but rather significantly reduced the binding to CLEC-2 (Figure 2A and 2B). Consistently with those Asp mutations, either mutation of Glu^47^ or Thr^52^ in PLAG3, and Glu^81^ or Thr^85^ in PLAG4 showed the reduction in the CLEC-2 binding (Supplementary Figure S2B). Thus, those three, Glu, Asp, and Thr are critical conserved residues for CLEC-2 binding in both PLAG domains. Importantly, the double mutant harboring D48A and D82A mutations almost completely lost the affinity to CLEC-2 (Figure 2B). These results were consistent with the results obtained using PLAG-deletion mutants (Figure 1C). Consistent with CLEC-2-binding activities, single mutations at Asp^48^ or Asp^82^ (D48A or D82A) also lowered the platelet-aggregating ability when compared with WT podoplanin (Figure 2C). Moreover, double mutant D48A/D82A could no longer exhibit platelet-aggregating ability (Figure 2C). These results indicate that PLAG4 plays a crucial role in podoplanin-induced platelet aggregation via the formation of a complex with CLEC-2.

### Establishment and characterization of anti-PLAG4 monoclonal antibodies

**Figure 3:**
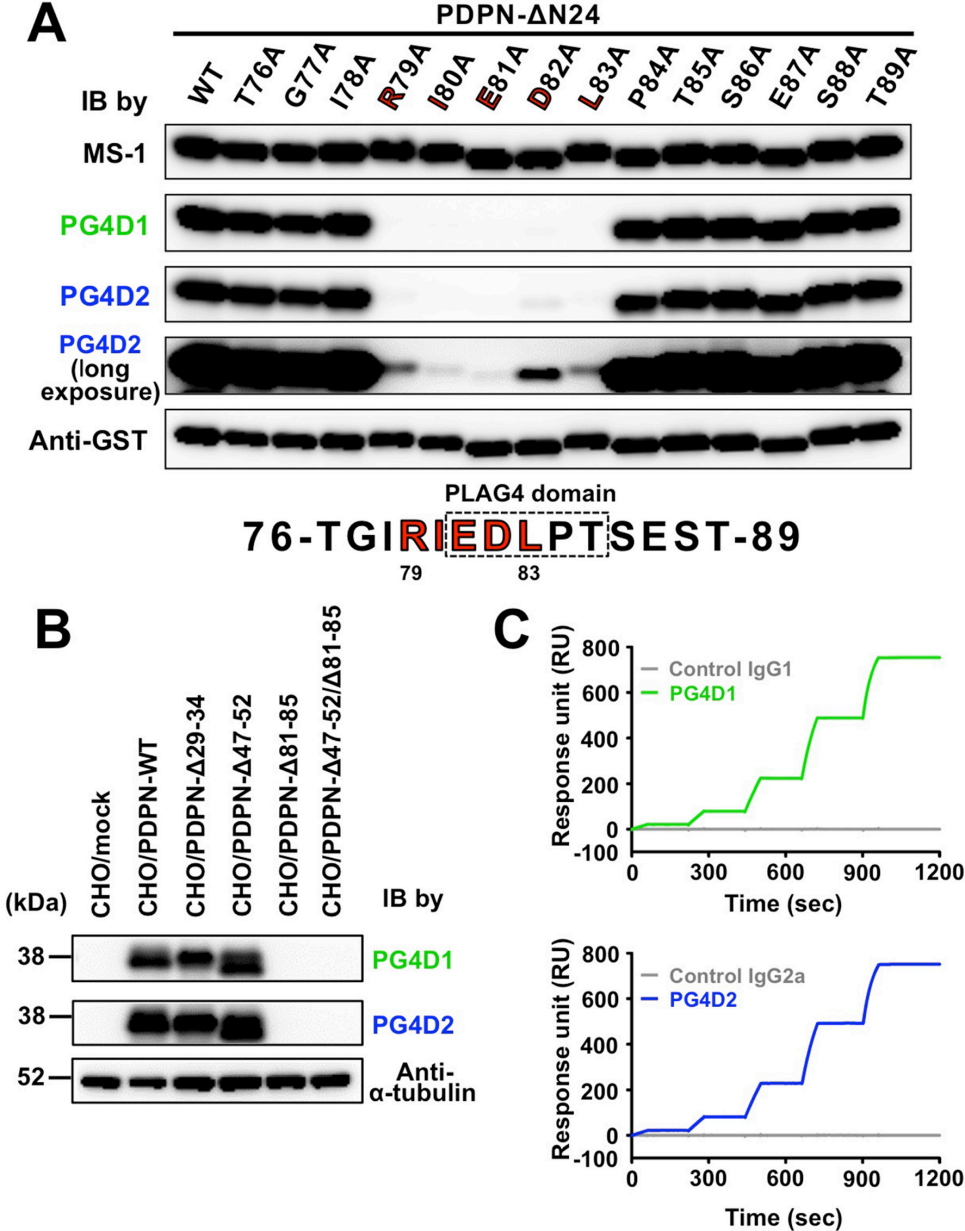
Establishment of PLAG4 domain-recognizing anti-podoplanin-neutralizing mAbs (**A**) GST-tagged recombinant human ΔN24-podoplanin protein (WT) and its point mutants were expressed in *E. coli.* Cell lysates were electrophoresed and immunoblotted with antibodies to podoplanin (MS-1, PG4D1, and PG4D2) or GST. In the fourth panel, the image of PG4D2 immunoblotting after long exposure is shown. The predicted PG4D1 and PG4D2 mAb-recognition domain (79-RIEDL-83) is indicated by red bold letters. (**B**) CHO cells that had been stably transfected with empty vector (mock), PDPN-WT, or PLAG domain-deleted PDPN mutants were lysed and immunoblotted with antibodies to podoplanin (PG4D1 and PG4D2) or α-tubulin. (**C**) Interaction between the human podoplanin protein immobilized on the CM-5 tip and control IgG1 (gray line, upper graph), the PG4D1 mAb (green line, upper graph), control IgG2a (gray line, lower graph), or the PG4D2 mAb (blue line, lower graph) was estimated by SPR analysis. Equilibrium dissociation constants (*K_D_*) of PG4D1 or PG4D2 mAbs on human podoplanin could not be calculated.

To further estimate the role of the PLAG4 domain in podoplanin-induced platelet aggregation and in pulmonary metastasis promoted by platelet aggregation, we attempted to establish a mouse mAb recognizing the PLAG4 domain. We purified a 12- or 40-times tandemly repeated human podoplanin PLAG4 peptide (TGIRIEDLPTSEST; 76–89 aa) as an antigen from a bacteria lysate and immunized the mice with the peptide. After fusing splenocytes with P3U1 myeloma cells, we screened hybridomas that secreted mAbs recognizing the immunogen, using enzyme-linked immunosorbent assay (ELISA)-based competition analysis. Upon further selection by recognition capability for podoplanin-expressing CHO/PDPN cells, but not for CHO/mock cells, we finally obtained two anti-PLAG4 mAbs, designated PG4D1 and PG4D2. The epitopes of PG4D1 and PG4D2 were identified by examining their reactivity to recombinant human ΔN24-podoplanin expressed in *E. coli* harboring point mutations of each amino acid to Ala (Ala scanning; Figure 3A). We confirmed that PG4D1 and PG4D2 mAbs recognized the perimeter structure from Arg^79^ to Leu^83^ (Figure 3A). Each amino acid from Arg^79^ to Leu^83^ was required for PG4D1 mAb recognition of human podoplanin. Within the recognition epitope, Arg^79^, Ile^80^, Glu^81^, and Leu^83^ residues were required for PG4D2 recognition to podoplanin, whereas the Asp^82^ residue appeared to be less effective than other amino acids because PG4D2 mAb could weakly recognize D82A mutant (Figure 3A and Supplementary Figure S3A). We confirmed that both mAbs specifically recognized the PLAG4 domain using PLAG deletion mutants (Figure 3B). The subclasses of PG4D1 and PG4D2 mAbs were identified to be IgG1 and IgG2a, respectively (data not shown). Surface plasmon resonance (SPR) analysis revealed that both mAbs exhibited a high affinity to human podoplanin (Figure 3C). The dissociation rate constant (*k*_d_) of both mAbs failed to reach values measurable by Biacore X100 due to the low level of dissociation, and so the precise equilibrium dissociation constant (*K*_D_) values could not be determined. However, the *K*_D_ values of both mAbs were less than 0.3 nM.

### Anti-PLAG4 mAbs PG4D1 and PG4D2 exhibit inhibitory activity against podoplanin-CLEC-2 binding, platelet aggregation, and pulmonary metastasis *in vivo*

**Figure 4:**
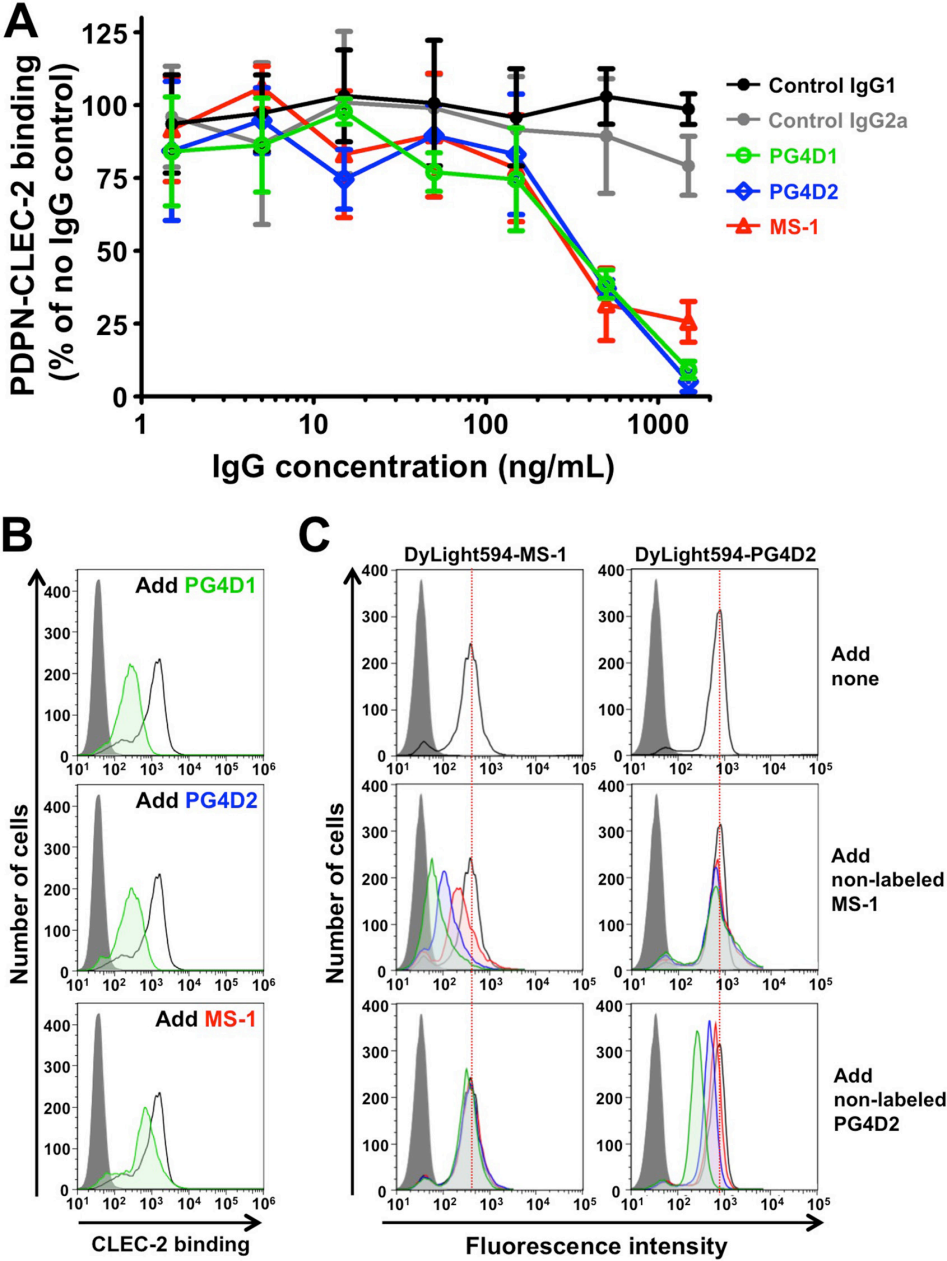
Neutralization of podoplanin–CLEC-2 binding by anti-PLAG4 mAbs PG4D1 and PG4D2 (**A**) Podoplanin-Fc protein (1 ng/well) was incubated with the indicated concentrations of control or anti-podoplanin antibodies in a 96-well plate. Next, CLEC-2-(His)_10_ protein (30 ng/well) was added to each well. After incubation with alpha screen beads, podoplanin-CLEC-2 binding intensity was measured as a fluorescence signal. No IgG control was taken as 100%. The graph shows mean ± SDs (*N* = 3). (**B**) CHO/PDPN-WT cells were incubated with 100 μg/mL of control IgG1/IgG2a or anti-podoplanin (PG4D1, PG4D2, or MS-1) antibodies, followed by incubation with 0.4 μg/mL of CLEC-2-(His)_10_ protein (open areas: control IgG-treated samples; green areas: PG4D1-, PG4D2-, or MS-1-treated samples). After washing, cells were further incubated with Alexa Flour 488-conjugated anti-penta-His second antibody. CLEC-2 binding was measured by flow cytometry. Grey areas indicate the fluorescence intensity of CLEC-2-non-treated samples. (**C**) CHO/PDPN-WT cells were incubated with 10 μg/mL of DyLight594-conjugated MS-1 or PG4D2 mAb (top panels). In some experiments, cold MS-1 or PG4D2 mAb (green areas: 10 μg/mL; blue areas: 3 μg/mL; red areas: 1 μg/mL) were co-treated with 10 μg/mL of DyLight594-conjugated antibodies (middle and bottom panels). After washing, fluorescence intensity was measured by flow cytometry.

To investigate the inhibitory activity of anti-PLAG4 mAbs against podoplanin–CLEC-2 interaction, we performed alpha screen-based competition analysis using recombinant human Podoplanin-Fc and CLEC-2-(His)_10_ proteins. The addition of each mAb to the reactants suppressed podoplanin-CLEC-2 interaction in a concentration-dependent manner (Figure 4A). At higher concentrations, PG4D1 and PG4D2 mAbs exerted stronger inhibitory activities than our previously generated MS-1 mAb, which targets the PLAG2/PLAG3 domain [23]. Consistently, and similar to the MS-1 mAb, masking the PLAG4 domain by PG4D1 and PG4D2 mAbs attenuated CHO/PDPN binding to recombinant CLEC-2 (Figure 4B). To exclude the possibility that anti-PLAG4 mAbs showed the inhibition of podoplanin-CLEC-2 binding by modulating the PLAG3-mediated binding to CLEC-2, we performed a competition assay again using the D48A/PLAG3 point mutant (Supplementary Figure S3B). PG4D1 and PG4D2 mAbs were able to neutralize CHO/PDPN-D48A binding to recombinant CLEC-2 in a dose-dependent manner and could almost completely suppress binding at the highest mAb concentration (100 μg/mL, Supplementary Figure S3B). To further confirm that PLAG3 and PLAG4 domains are independently associated with CLEC-2, we carried out competitive flow cytometric analyses using fluorescent dye (DyLight594)-labeled PG4D2 and MS-1 mAbs (Figure 4C). The reaction of DyLight594-labeled MS-1 mAb to CHO/PDPN was attenuated by the preincubation of the cells with non-labeled MS-1 mAb but not by the non-labeled PG4D2 and vice versa (Figure 4C). These results indicated that PG4D2 and MS-1 mAbs did not sterically hinder the mutual targeting PLAG domain and that masking one PLAG domain did not induce conformational changes affecting CLEC-2 interaction through the other PLAG domain. Therefore, PLAG3 and PLAG4 domains would independently bind to CLEC-2.

**Figure 5:**
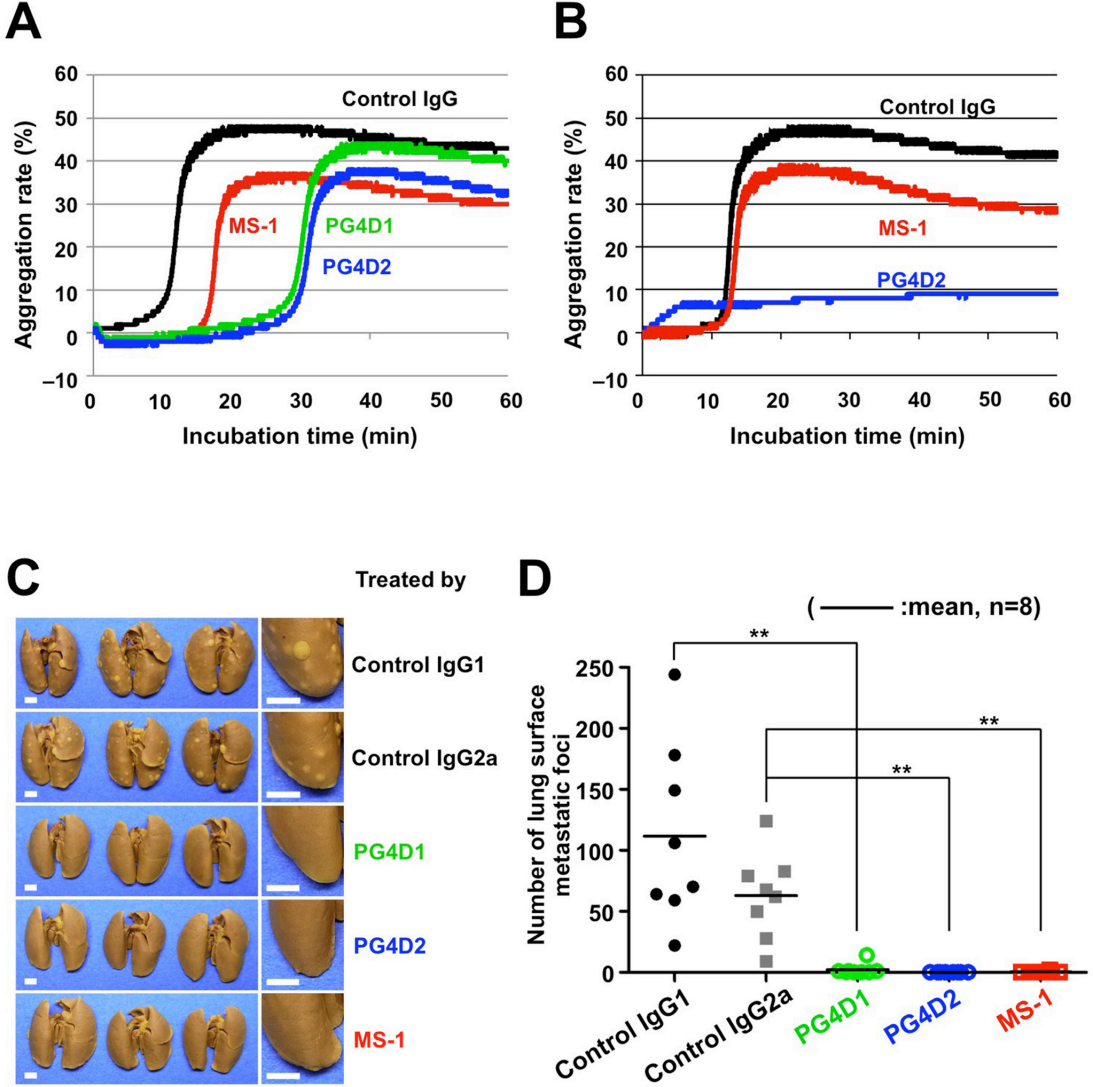
Inhibition of podoplanin-mediated platelet aggregation and pulmonary metastasis by anti-PLAG4 mAbs PG4D1 and PG4D2 (**A** and **B**) CHO/PDPN-WT cells (A) or CHO/PDPN-D48A cells (B) were incubated with 10 μg/mL of the indicated antibodies followed by incubation with mouse PRP. The aggregation rate was estimated using an aggregometer. (**C** and **D**) BALB/c-*nu/nu* mice were intravenously injected with 10 μg/mouse of the indicated antibodies. The next day, CHO/PDPN-WT cells (2.5 × 10^5^ cells/mouse) were intravenously inoculated into the mice. After 19 days of tumor inoculation, the mice were euthanized, and lung surface metastatic foci were counted. The representative pictures of lungs are shown (C). Areas surrounded by boxes are magnified (right pictures). Bars, 2 mm. The number of metastatic foci is shown (D). Bars, mean (*N* = 8). **P < 0.01 using the Mann–Whitney *U* test.

We previously reported that podoplanin–CLEC-2 interaction was essential for podoplanin-induced platelet aggregation and tumor metastasis [3, 6]. We therefore examined the effect of anti-PLAG3 mAb MS-1 and anti-PLAG4 mAbs PG4D1 and PG4D2 on podoplanin-induced platelet aggregation. The incubation of mouse platelets with CHO/PDPN cells induced platelet aggregation, and the aggregation starting time was delayed by adding MS-1, PG4D1, or PG4D2 mAb (Figure 5A). The platelet aggregation induced by D48A/PLAG3-mutated CHO/PDPN cells could be suppressed almost completely by adding anti-PLAG4 mAb PG4D2 but not by MS-1 mAb (Figure 5B). Similarly, the platelet aggregation induced by D82A/PLAG4-mutated CHO/PDPN cells could be almost completely suppressed by adding anti-PLAG3 mAb MS-1 but not by PG4D2 mAb (Supplementary Figure S4). These results demonstrated that the PLAG4 domain possesses platelet aggregation ability in addition to PLAG3 domain in human podoplanin. We next performed an *in vivo* metastasis assay to verify the role of the PLAG4 domain. Podoplanin-induced pulmonary metastasis was significantly blocked by a single administration of PG4D1 or PG4D2 mAb on the day before tumor inoculation, like MS-1 mAb (Figure 5C and 5D). These results indicate that the PLAG4 domain has a role in podoplanin-mediated metastasis formation, and blocking the interaction between PLAG4–CLEC-2 would be a promising strategy for suppressing hematogenous metastasis.

## DISCUSSION

We previously identified podoplanin/Aggrus/gp44 as a novel platelet aggregation-inducing factor and then investigated the domain critical to its platelet aggregation-inducing activity [6]; we discovered that the EDXXVTPG motif is associated with its platelet-aggregating activity, therefore designated the domain as the PLAG domain [6]. Almost all mammalian podoplanins contain three tandem repeats of the PLAG domain at the extracellular domain (PLAG1–3) [12]. Consistent with our previous finding that *O*-glycanase treatment attenuated the platelet-aggregating activity of podoplanin/gp44 [13], the Thr residue within the PLAG domain was found to be *O*-glycosylated [25], and the *O*-glycosylation is indispensable for exhibiting its platelet-aggregating activity. This was evidenced by the fact that substitution of Thr^52^ to Ala in the human PLAG3 domain (47-EDDVVT^52^PG-54) attenuated the activity and that podoplanin expressed in glycosylation-deficient CHO mutants, Lec-2 or Lec-8, exhibited no platelet-aggregating activity [6, 14]. Moreover, a synthetic podoplanin peptide containing human PLAG2 and PLAG3 domains exhibited platelet-aggregating activity only after *O*-glycosylation at the Thr^52^ residue [15]. A recent report on crystal structure revealed that *O*-glycans attached to Thr^52^ in addition to Glu^47^ and Asp^48^ residues in the human PLAG3 domain are associated with the binding to Arg residues in CLEC-2 [24]. Thus, the minimum structure essential for the binding to CLEC-2 appeared to be two negatively charged amino acids following an *O*-glycosylated amino acid (i.e., EDXXXT). In the case of our newly identified PLAG4 domain (81-EDLPT^85^SE-87), Thr^85^ and Ser^86^ residues appeared to be *O*-glycosylated since the NetOGlyc 4.0 Server [26] predicted a higher possibility of *O*-glycosylation at the Thr^85^ and Ser^86^ residues in the human PLAG4 domain than at the Thr^52^ residue in the human PLAG3 domain. Moreover, Zimmer et al. previously suggested glycosylation at the Thr^92^ residue within the canine PLAG4 domain (88-EDGPT^92^QE-94) [25]. Therefore, *O*-glycosylated Thr^85^ and/or Ser^86^ in addition to negatively charged Glu^81^ and Asp^82^ residues in the human PLAG4 domain would be associated with the binding to CLEC-2 and the platelet-aggregating activity of podoplanin. Based on the low homology of the Ser^86^ residue among mammalian podoplanin, *O*-glycans attached to the Ser^86^ residue in the human PLAG4 domain may not be required for CLEC-2 binding but may contribute to robust PLAG4–CLEC-2 interaction. At least, we indicated the contribution of Thr^85^ residue to CLEC-2 binding using its Ala-substituted mutant (Supplementary Figure S2B). The number of amino acids intervening between negatively charged Glu–Asp residues and *O*-glycosylated Thr residue is two in the PLAG4 domain and three in the PLAG1–3 domains (Figure 1E). Moreover, the EDXXT motif in the PLAG4 domain is highly conserved among mammals (36/42 species from multiple sequence alignment analysis; Supplementary Figure S1), similar to the EDXXXT motif in PLAG1 and PLAG3 (40/42 and 38/42 species, respectively). Therefore, we find it appropriate to revise the PLAG consensus sequence to ED(X)XXT, as shown in Figure 1E.

The substitution of positively charged Arg residues in CLEC-2 has been reported to suppress the binding to podoplanin almost completely [24]. Although the deletion of the PLAG3 or PLAG4 domain weakened this interaction, the deletion of both PLAG3 and PLAG4 domains resulted in a complete loss of podoplanin–CLEC-2 interaction (Figure 1C). Furthermore we have confirmed Glu^81^, Asp^82^, and Thr^85^ residues in the PLAG4 domain, with the equivalent positions of Glu^47^, Asp^48^, and Thr^52^ residues in the PLAG3 domain, as essential for CLEC-2 binding (Figure 2B and Supplementary Figure S2B). These results suggest that the human PLAG4 and PLAG3 domains share a binding site on CLEC-2. Because podoplanin could coincidentally form a complex with an antibody (150 kDa) larger than CLEC-2 (35 kDa), (Figure 4C), it is possible that one molecule of podoplanin could bind to two molecules of CLEC-2 at the same time. This possibility is an advantage for Src and Syk signal activation via a CLEC-2 dimer [27]. Two CLEC-2-binding sites located on the podoplanin molecule may contribute to CLEC-2 clustering and to fast signal transduction [28]. Although the monomer podoplanin-Fc protein alone could not induce platelet aggregation, the addition of a secondary antibody that enhanced CLEC-2 crosslinking and oligomerization could trigger platelet aggregation (data not shown). These results suggest that the condensation of CLEC-2 in the cell–platelet adhesion area would be essential for transmitting platelet-aggregating signals via CLEC-2.

The actual role sharing between the PLAG3 and PLAG4 domains in CLEC-2 binding remains unknown. PLAG4 deletion or PLAG4 mutation resulted in a drastic decrease in podoplanin–CLEC-2 binding when compared with PLAG3 deletion or PLAG3 mutation (Figure 1C and 2B). Moreover, the anti-PLAG4 mAbs (PG4D1 and PG4D2) exerted stronger inhibitory activity against podoplanin–CLEC-2 binding than did the anti-PLAG3 mAb (MS-1) did (Figure 4A). These results suggest the importance of the PLAG4 domain in podoplanin–CLEC-2 binding. However, we could not exclude the possibility that each cell uses the PLAG3 and PLAG4 domains according to cell context or microenvironment. In fact, the anti-PLAG3 mAb preferentially recognized podoplanin expressed on H226, PC-10, and KYSE70 cells, whereas anti-PLAG4 mAbs preferentially recognized podoplanin expressed on UM-UC-5 and HT1080 cells (Supplementary Figure S5). As no mutation was found in podoplanin expressed in these cell lines, post-translational modifications, i.e., glycosylation, and interaction with other molecules, etc. may affect the reactivity of the mAbs. These notions were supported by a recent report that the anti-PLAG4 mAb preferentially recognized metastasized osteosarcomas [29].

Our established anti-PLAG4 neutralizing mAbs, PG4D1 and PG4D2, exhibited a high affinity to human podoplanin with very low *K*_D_ values (less than 0.3 nM). The single administration of the anti-PLAG4 mAbs into mice could almost completely suppress hematogenous metastasis of CHO/PDPN cells. Since a PG4D1 mAb of murine IgG1 subclass exhibiting low ADCC/CDC activity and a PG4D2 mAb of murine IgG2a subclass having strong ADCC/CDC activity could suppress pulmonary metastasis, ADCC/CDC activity would not be associated with the metastasis-inhibitory function of the mAbs, similarly to the anti-PLAG3 mAb MS-1 [23]. Considering the complete metastasis suppression by only a single administration of anti-PLAG3 mAb MS-1 (Figure 5C and 5D) [23], the transient suppression of podoplanin–CLEC-2 binding would be sufficient to suppress podoplanin-mediated hematogenous metastasis. With regard to tumor suppression, ADCC/CDC activity may be partly involved in the anti-tumor activity of the anti-podoplanin antibodies because an anti-PLAG3 mAb MS-1 of murine IgG2a subclass having strong ADCC/CDC activity, but not an anti-PLAG3 mAb P2–0 of murine IgG1 subclass exhibiting low ADCC/CDC activity, could attenuate CHO/PDPN proliferation in nude mice [23]. Of course, the inhibition of podoplanin–CLEC-2 binding would be sufficient to suppress the proliferation of some tumors *in vivo* [23]. However, the interruption of mAb administration resulted in recurrence of the tumors [23]. These results indicate that either the use of mAb possessing strong ADCC/CDC activity or the complete inhibition of podoplanin–CLEC-2 binding by the combined administration of anti-PLAG3 and anti-PLAG4 mAbs may be required for inducing complete tumor remission. As podoplanin is known to be expressed in normal tissue, including lymphatic vessels, kidney podocytes, mesothelium, and alveolar epithelium, the complete blockade of podoplanin–CLEC-2 binding by the co-administration of anti-PLAG3 and anti-PLAG4 mAbs possessing no ADCC/CDC activity would be preferable as anti-tumor agents with minimal side effects.

In this report, we succeeded in identifying the novel PLAG4 domain that is associated with the binding to CLEC-2 and platelet aggregation. Based on the PLAG4 domain sequence, we would propose ED(X)XXT as the revised PLAG domain consensus sequence. Our finding is expected to shed light on the clinical development of podoplanin-targeting cancer therapy.

## MATERIALS AND METHODS

### Cells and cell culture conditions

CHO cells were purchased from the American Type Culture Collection (ATCC, Manassas, VA, USA) and cultured in PRMI 1640 medium (Wako, Osaka, Japan) containing 10% FBS (Sigma-Aldrich, St Louis, MO, USA). Stable podoplanin-expressing clones were established as described previously [6] and cultured in a medium containing 250 μg/mL of G418 (Life Technologies, Carlsbad, CA, USA).

### Plasmid construction

The pcDNA3 vector containing WT human *podoplanin* cDNA (pcDNA3-*podoplanin*) was established as described previously [6]. pcDNA3 vectors containing *podoplanin* deletion mutated cDNAs (PDPN-Δ29–34, PDPN-Δ47–52, PDPN-Δ81–85, and PDPN-Δ47–52/Δ81–85) or *podoplanin* point-mutated cDNAs (PDPN-G45A, PDPN-D48A, PDPN-D48N, PDPN-D48E, PDPN-D49A, PDPN-D82A, and PDPN-D48A/D82A) were generated from pcDNA3-*podoplanin* as a template using the QuikChange site-directed mutagenesis kit (Agilent Technology, Santa Clara, CA, USA). pGEX-6P-3 vectors (GE Healthcare, Buckinghamshire, UK) containing signal peptide-deleted *podoplanin* cDNA (PDPN-ΔN24) or its point-mutated cDNAs were constructed as described previously [22].

### Animals

Female BALB/c, BALB/c-*nu/nu*, and Jcl:ICR mice were purchased from Charles River (Kanagawa, Japan). All animal procedures were performed according to the protocols approved by the Japanese Foundation for Cancer Research Animal Care and Use Committee.

### Multiple sequence alignment and window analysis

All podoplanin protein sequences were obtained from NCBI's reference sequence database (http://www.ncbi.nlm.nih.gov/refseq/). For the alignment of podoplanin sequences, sequences including multiple gaps were excluded, and typical experimental animal species and representative species of each family were selected. The selected 42 sequences are presented as Supplementary Information (Supplementary Figure S1). Sequences were aligned using the muscle alignment algorithm available in the MEGA6 software [30]. Sliding-window analysis was performed using the AL2CO program [31]. Hydropathy plots were generated using Kyte and Doolittle amino acid hydropathy scores [32].

### Immunoblotting

Cells were lysed in TENSV buffer (50 mM Tris-HCl [pH 7.5], 100 mM NaCl, 2 mM EDTA, 1% NP-40, 0.1% aprotinin, and 2 mM phenylmethylsulfonyl fluoride) and electrophoresed followed by the transfer to a PDVF membrane (Millipore, Billerica, Massachusetts, USA). The GST-tagged human PDPN-ΔN24 and its point mutants were produced in BL21 (DE3) *E. coli* (Thermo Fisher Scientific, Waltham, MA, USA), and the cell pellets were boiled in SDS sample buffer for immunoblots. The membranes were incubated with antibodies to podoplanin (D2–40, AbD Serotec, Oxford, UK; FL-162, Santa Cruz Biotechnology, Santa Cruz, CA, USA; MS-1; P2–0; PG4D1; and PG4D2), α-tubulin (Sigma-Aldrich, St Louis, MO, USA), and GST (Abcam, Cambridge, UK).

### Flow cytometry

Cells were harvested and treated with 1 μg/ml of anti-podoplanin antibodies or control mouse IgG (Sigma-Aldrich, St Louis, MO, USA), following incubation with 4 μg/mL of Alexa Flour 488-conjugated anti-mouse IgG (H + L) (Thermo Fisher Scientific, Waltham, MA, USA). For CLEC-2-binding analysis, cells were incubated with 0.4 μg/mL of (His)_10_-tagged human CLEC-2 protein (R & D Systems, Minneapolis, MN, USA), following incubation with 1/500 dilutions of Alexa Flour 488-conjugated anti-penta-His antibody (QIAGEN, Venlo, Netherlands). Flow cytometric analyses were performed using Cytomics FC500 flow cytometry system (Beckman Coulter, CA, USA). In some experiments, MS-1 and PG4D2 mAbs were used as primary antibodies, following the conjugation of DyLight594 using the DyLight antibody labeling kit (Thermo Fisher Scientific, Waltham, MA, USA).

### Platelet aggregation assay

Murine whole blood was drawn by cardiac puncture from Jcl:ICR mice terminally anesthetized with sevoflurane and taken with heparin solution. Platelet-rich plasma (PRP) was obtained from the supernatant of whole blood by centrifugation at 120 x*g* for 8 min twice. Platelets were collected from the pellets of PRP by centrifugation at 500 x*g* for 10 min, and the supernatant was stocked as platelet-poor plasma (PPP). Washed platelets were prepared by washing the pellets with the modified Tyrode's buffer (137 mM NaCl, 11.9 mM NaHCO_3_, 0.4 mM Na_2_HPO_4_, 2.7 mM KCl, 1.1 mM MgCl_2_, and 5.6 mM glucose, pH 7.3) followed by centrifugation under the same conditions and resuspended in the modified Tyrode's buffer at a concentration of 1 × 10^8^/mL. Prior to the assay, 10% PPP and 400 μM CaCl_2_ were supplied to the washed platelets. Next, the washed platelets were incubated with cell suspension (1 × 10^8^ cells/mL). For the antibody inhibition assay, cells were incubated with 10 μg/mL of mAb or control IgG for 30 min on ice before incubation with platelets. The platelet aggregation rate was examined using a platelet aggregometer (MCM HEMA TRACER 313M; SSR Engineering, Kanagawa, Japan).

### Hybridoma production and antibody purification

A human *podoplanin* cDNA region encoding amino acids 76–89 (226–267 bp) was cloned and tandemly connected 12 and 40 times. These cDNA fragments were inserted into a pGEX-6P-3 vector (GE Healthcare, Buckinghamshire, UK). Next, the GST-tagged human podoplanin peptide (76–89 aa) produced in BL21 (DE3) *E. coli* was purified using glutathione sepharose. Six-week-old female BALB/c mice were injected with the GST-tagged peptide as an immunogen in conjugation with Titer MAX Gold adjuvant (Titer MAX, Norcross, GA, USA). Further, intraperitoneal immunization was performed intermittently for two months. Mice were euthanized, and splenocytes were fused with mouse myeloma P3U1 cells using PEG4000 (Merck, Whitehouse Station, NJ, USA). Hybridoma screening and antibody purification from ascites were performed as described previously [22]. IgG isotypes were identified using the Mouse Monoclonal Antibody Isotyping Test Kit (AbD Serotec, Oxford, UK).

### SPR analysis

SPR analysis was performed using Biacore X100 (GE healthcare, Buckinghamshire, UK). Recombinant human podoplanin-Fc protein (R & D Systems, Minneapolis, MN, USA) was immobilized on a CM5 sensor chip (GE Healthcare, Buckinghamshire, UK). Final levels of immobilization were approximately 562.2 or 562.6 response units (measure conditions of PG4D1 or PG4D2, respectively). Five concentrations of PG4D1, PG4D2 mAbs, and their control mouse IgGs were flowed over the chip in the single cycle kinetics. Sensorgrams were fit by global analysis using the Biacore X100 evaluation software. Efforts toward determining the equilibrium dissociation constant (*K*_D_) were decided using the bivalent binding analyte model.

### Alpha screen

Podoplanin-Fc protein (1 ng/well) was firstly incubated with PG4D1, PG4D2, and MS-1 mAbs, or control IgG1 and IgG2a in a 96-well plate. Next, (His)_10_-tagged human CLEC-2 protein (30 ng/well) was added to the mixture, following the addition of alpha screen beads (Nickel Chelate Donor beads and Protein A Acceptor beads; Perkin Elmer, Waltham, MA, USA). After incubation for 30 min at RT, binding intensity was measured as a fluorescence signal using the EnVision plate reader (Perkin Elmer, Waltham, MA, USA).

### Experimental pulmonary metastasis

Five-week-old female BALB/c-*nu*/*nu* mice were intravenously inoculated with antibodies (10 μg/mouse) the day before cell injection. CHO/PDPN cells were resuspended in Hanks’ Balanced Salt Solution (HBSS, Gibco, Thermo Fisher Scientific, Waltham, MA, USA) and intravenously injected into mice (2.5 × 10^5^ cells/mouse). Eighteen days following CHO/PDPN injection, the mice were euthanized, and their lungs were stained with the saturated picric acid solution. Surface lung metastatic foci were counted.

### Statistical analysis

A Mann–Whitney *U* test was performed to determine the statistical significance in flow cytometry and metastasis assays. Significant probability (*P*) values are shown as *P < 0.05, and **P < 0.01. All statistical tests were two-sided.

## SUPPLEMENTARY MATERIAL FIGURES AND TABLE




