# Supplementary material for: Targeting a novel domain in podoplanin for inhibiting platelet-mediated tumor metastasis
Source: Oncotarget. 2015 Dec 14;7(4):3934–46. doi: 10.18632/oncotarget.6598 (PMC4826181; doi:10.18632/oncotarget.6598)
Supplement: Supplementary file 1 [file oncotarget-07-3934-s001.pdf]

Targeting a novel domain in podoplanin for inhibiting platelet-mediated tumor metastasis

Supplementary Materials

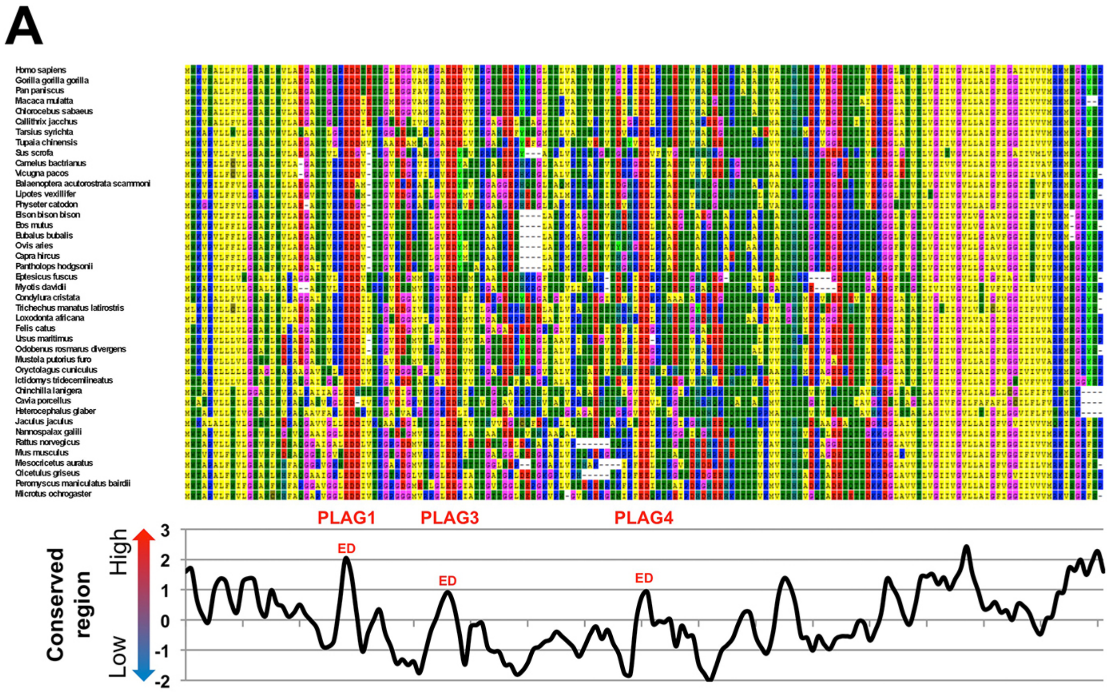

B

Binomial name	Accession No.	Binomial name	Accession No.
Homo sapiens	NP_006465.3	Myotis davidii	XP_006766770.1
Gorilla gorilla gorilla	XP_004024751.1	Condylura cristata	XP_004679390.1
Pan paniscus	XP_003806315.1	Trichechus manatus latirostris	XP_004377346.1
Macaca mulatta	XP_001106933.2	Loxodonta africana	XP_010591406.1
Chlorocebus sabaeus	XP_007978670.1	Felis catus	XP_006934362.1
Callithrix jacchus	XP_003733393.2	Ursus maritimus	XP_008692925.1
Tarsius syrichta	XP_008057741.1	Odobenus rosmarus divergens	XP_004397188.1
Tupaia chinensis	XP_006145340.1	Mustela putorius furo	XP_004783770.1
Sus scrofa	XP_005665074.1	Oryctolagus cuniculus	XP_008273589.1
Camelus bactrianus	XP_010955812.1	Ictidomys tridecemlineatus	XP_005317538.1
Vicugna pacos	XP_006197091.1	Chinchilla lanigera	XP_005409948.1
Balaenoptera acutorostrata scammoni	XP_007174997.1	Cavia porcellus	XP_005004460.1
Lipotes vexillifer	XP_007463244.1	Heterocephalus glaber	XP_004850371.1
Physeter catodon	XP_007104886.1	Jaculus jaculus	XP_004649668.1
Bison bison bison	XP_010848362.1	Nannospalax galili	XP_008854276.1
Bos mutus	XP_005889851.1	Rattus norvegicus	NP_062231.1
Bubalus bubalis	XP_006074521.1	Mus musculus	NP_034459.2
Ovis aries	XP_004013851.1	Mesocricetus auratus	XP_005081155.1
Capra hircus	XP_005690878.1	Cricetulus griseus	XP_007606119.1
Pantholops hodgsonii	XP_005966349.1	Peromyscus maniculatus bairdii	XP_006975508.1
Eptesicus fuscus	XP_008146423.1	Microtus ochrogaster	XP_005352936.1

Supplementary Figure S1: Alignment of 42 mammalian podoplanin protein sequences. (**A** and **B**) All podoplanin protein sequence data was obtained from NCBI Reference Sequence Database (http://www.ncbi.nlm.nih.gov/refseq/) on February 13, 2015 and aligned using the muscle alignment algorithm available in the MEGA6 software. The aligned multi-sequence was generated by repeated alignments eliminating the gaps existed against Homo sapiens podoplanin. All sequences used for the ultimate alignment are listed in Supplementary Table S1. The conserved region was analyzed using sliding-iens window analysis.

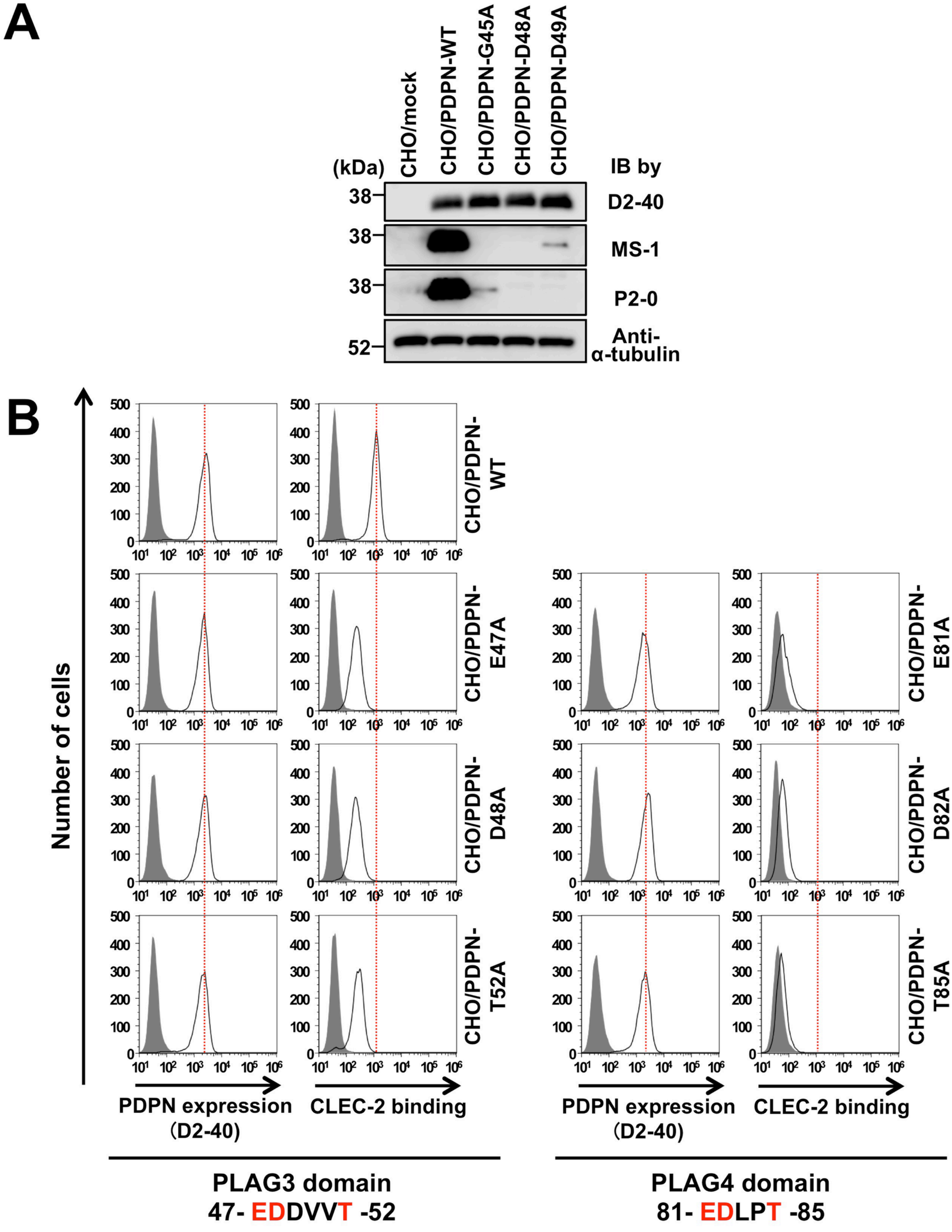

Supplementary Figure S2: Every conserved residue Glu, Asp, and Thr in PLAG3/4 domains is essential for podoplanin-CLEC-2 interaction. (**A**) CHO cells that had been stably transfected with empty vector (mock), PDPN-WT, or PLAG3 point mutants were lysed and immunoblotted with the indicated antibodies. (**B**) CHO cells that had been stably transfected with PDPN-WT, PLAG3 point mutants (E47A, D48A, and T52A), or PLAG4 point mutants (E81A, D82A, and T85A) were treated with control mouse IgG (closed areas) or anti-PDPN mAb (D2–40; open areas) for examining PDPN expression level (left panels), or with PBS (closed areas) or CLEC-2-$(His)_{10}$ (open areas) for estimating CLEC-2-binding ability (right panels). After washing, cells were incubated with Alexa Flour 488-conjugated second antibody.

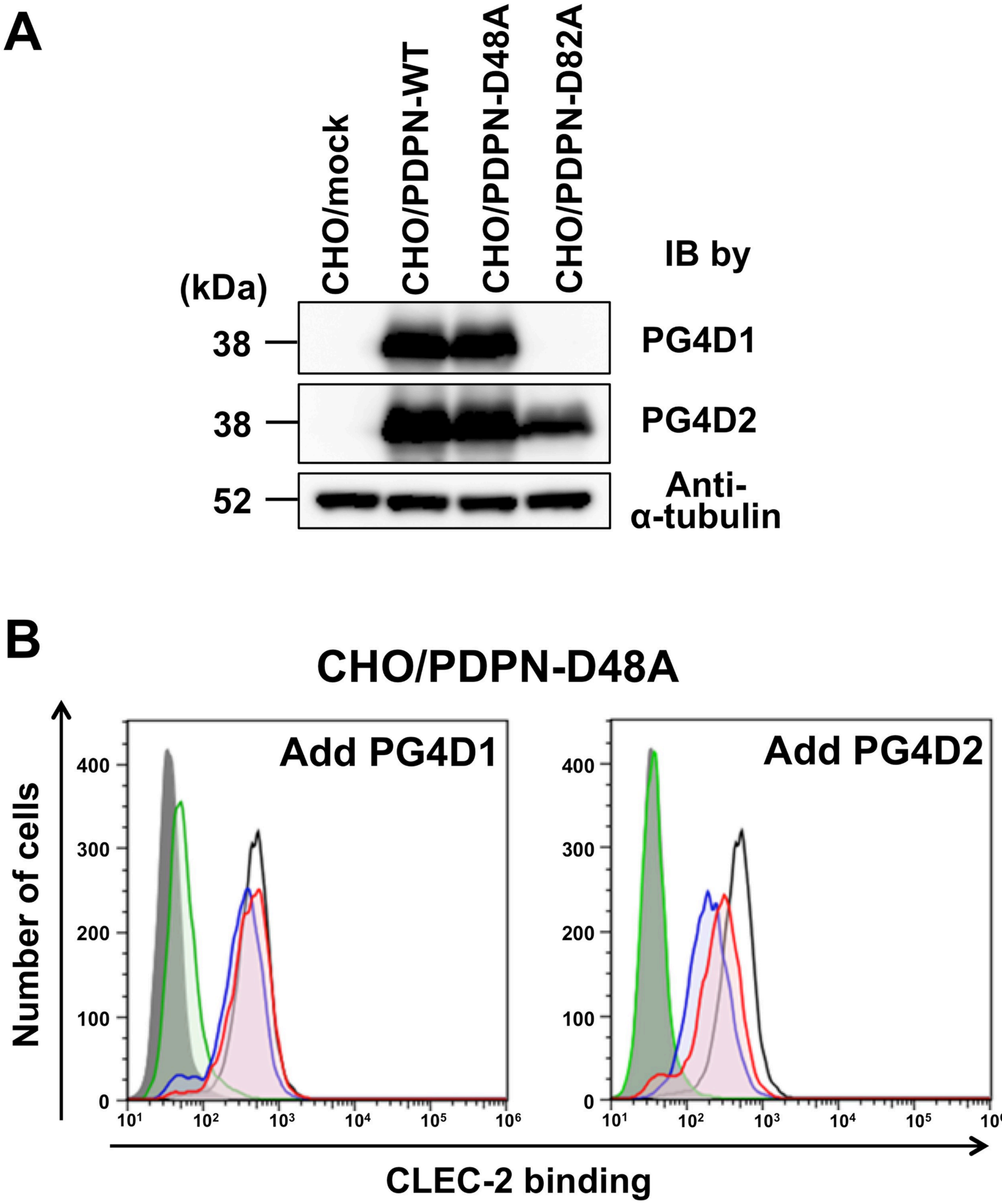

Supplementary Figure S3: Neutralization of PLAG3-mutated podoplanin binding to CLEC-2 by anti- PLAG4 mAbs PG4D1 and PG4D2. (**A**) CHO cells that had been stably transfected with empty vector (mock), PDPN-WT or PLAG3/4 point mutants were lysed and immunoblotted with the indicated antibodies. (**B**) CHO/PDPN- D48A cells were first incubated with 100 µg/mL of control IgGl (left panel, closed area) or control IgG2a (right panel, closed area). In some experiments, cells were preincubated with 100 µg/mL (green area), 10 µg/mL (blue area), 1 µg/mL (red area), or 0 µg/mL (open area) of PG4D1 mAb (left panel) or PG4D2 mAb (right panel), followed by addition of 0.4 µg/mL of CLEC-2-$(His)_{10}$. After washing, cells were further incubated with Alexa Flour 488-conjugated anti-penta- His second antibody. CLEC-2 binding was measured by flow cytometry.

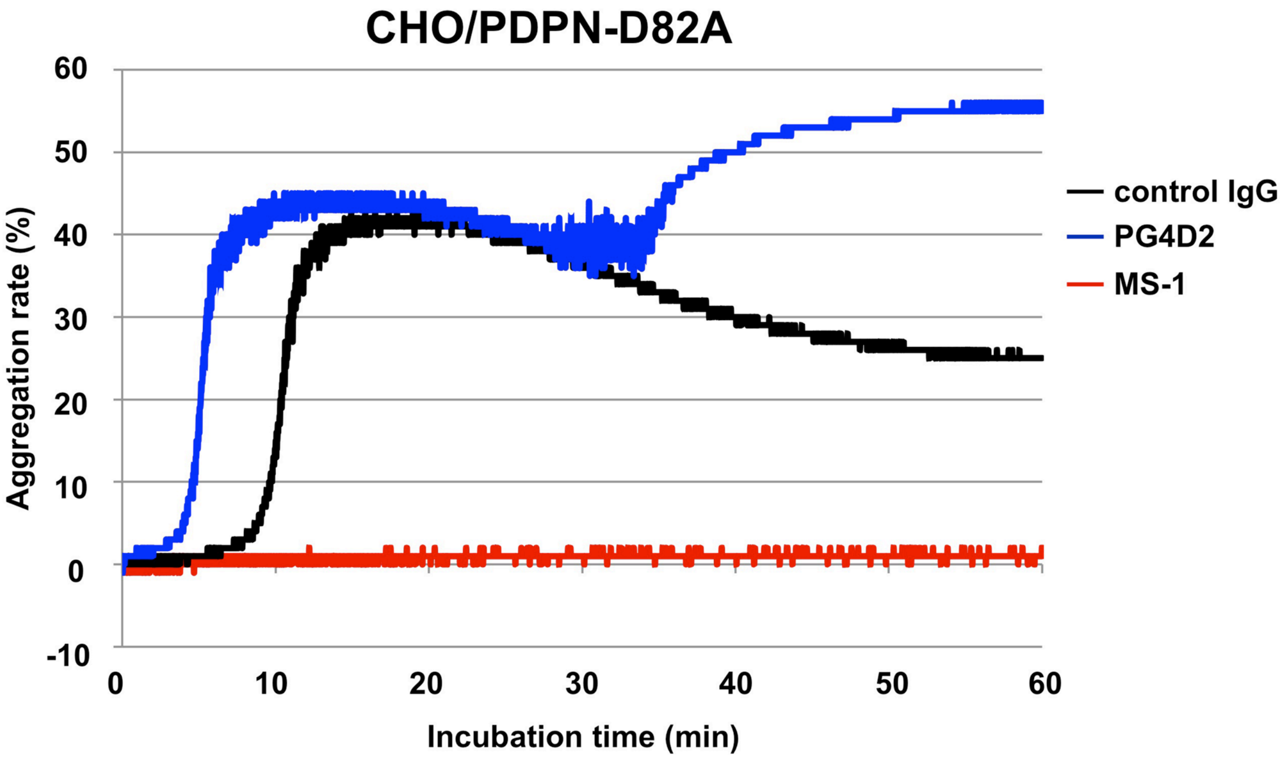

Supplementary Figure S4: Complete inhibition of podoplanin-D82A mutant-mediated platelet aggregation by anti-PLAG3-neutralizing mAb MS-1. Although the PLAG4 domain is associated with CLEC-2 binding, mutation within the PLAG4 domain could not completely suppress the podoplanin-mediated platelet aggregation (control IgG and PG4D2). Blockade of PLAG3 function by anti-PLAG3 neutralizing mAb MS-1 almost completely suppressed the podoplanin-mediated platelet aggregation, indicating the importance of PLAG3 and PLAG4 domains both being associated with platelet-aggregating ability of human podoplanin.

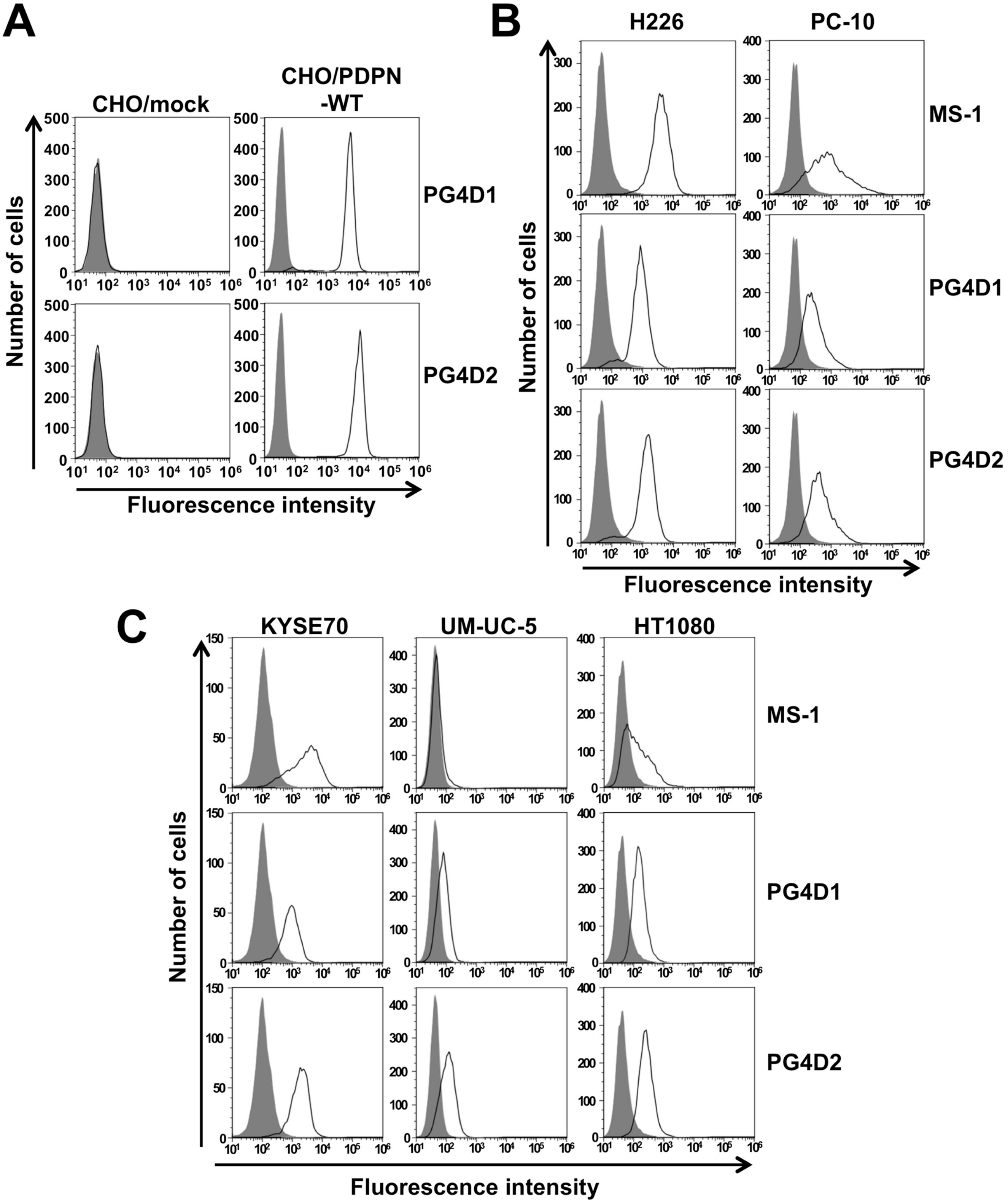

Supplementary Figure S5: Recognition of endogenous human podoplanin by PG4D1 and PG4D2 mAbs. (**A**) CHO cells that had been stably transfected with empty vector (mock) or PDPN-WT were treated with 1 µg/mL of control IgG (closed areas), PG4D1 (top panels), and PG4D2 (bottom panels) mAbs on ice. After incubation with Alexa Flour 488-conjugated second antibody, podoplanin expression was measured by flow cytometry. (**B** and **C**) NCI-H226, PC-10, KYSE70, UM-UC-5, and HT1080 cells were treated with control IgG (closed areas), MS-1 (top panels), PG4D1 (middle panels), and PG4D2 (bottom panels) mAbs. Podoplanin expression and reactivity was measured as in (A).
